# Neutrophils in cancer: prognostic role and therapeutic strategies

**DOI:** 10.1186/s12943-017-0707-7

**Published:** 2017-08-15

**Authors:** Alberto Ocana, Cristina Nieto-Jiménez, Atanasio Pandiella, Arnoud J Templeton

**Affiliations:** 1 Medical Oncology Department, Albacete University Hospital and Translational Research Unit, Albacete, Spain; 2 Centro de Investigación del Cáncer CSIC and CIBERONC, Salamanca, Spain; 3 Division of Medical Oncology, St. Claraspital Basel and Faculty of Medicine, University of Basel, Basel, Switzerland

**Keywords:** Neutrophils, Neutrophil-lymphocyte ratio, Prognosis, Target

## Abstract

Expression of high levels of immune cells including neutrophils has been associated with detrimental outcome in several solid tumors and new strategies to decrease their presence and activity are currently under clinical development. Here, we review some of the relevant literature of the role of neutrophils in different stages of the oncogenic process including tumor initiation, growth, proliferation or metastatic spreading and also focus on how neutrophil counts or the neutrophil-to-lymphocyte ratio may be used as a prognostic and predictive biomarker. Strategies to avoid the deleterious effects of neutrophils in cancer and to reduce their activity are discussed. Examples for such strategies include inhibition of CXCR1 and CXCR2 to decrease migration of neutrophils to tumoral areas or the inhibition of granulocyte colony stimulating factor to decrease the amount of neutrophils which has shown efficacy in preclinical models.

## Background

Different strategies have been explored and developed in the fight against cancer. Classically, therapies have been designed against molecular alterations that drive the transformation of normal cells into tumor ones [1]. This approach has been successful and agents against oncogenic alterations like those targeting HER2 overexpression in breast and gastric cancer, or BRAF in melanoma, have shown clinical benefit [1]. Recently, drugs that boost the host immune system, like those targeting immunologic checkpoints, have shown promising activity in different solid tumors [2]. Activation of cytotoxic T lymphocytes by avoiding host immunotolerance has demonstrated utility when using CTLA4, PD1, and PD-L1 inhibitors [2]. However, other potential immunologic targets could be exploited therapeutically. It is known that different cells participate in the immune response against cancer making this process dynamic, where a balance between activating and repressing signals takes place. Recently, the role of neutrophils in cancer has attracted attention. Expression of high levels of these cells has been associated with detrimental outcome in several solid tumors and new strategies to decrease their presence and activity are currently in clinical development [3–6].

In this brief review we summarize some of the relevant data that associates neutrophils with cancer. We will focus on how neutrophil counts could be used as a prognostic and predictive biomarker and how therapeutic agents against them are reaching the clinical development stage.

### The biology of neutrophils: Clinical implications

Neutrophilic granulocytes (neutrophils) account for 50–70% of all leukocytes and depend on a sequential process of maturation in the bone marrow that provokes the conversion of myeloblasts to segmented neutrophils [7]. Maturation depends on different stimulating factors including the granulocyte–macrophage-colony stimulating factor (GM-CSF) and the granulocyte-colony stimulating factor (G-CSF), two of the most relevant growth factors that control such maturation process. Neutrophil maturation includes: myeloblast, promyelocyte, myelocyte, metamyelocyte, band neutrophil and, finally, segmented neutrophils [7–9]. Neutrophil lifespan is altered in cancer and it is associated with maturation, extending from 7 h in normal conditions to 17 h in cancer [8, 9]. Of note, the majority of neutrophils remain in the bone marrow, for instance in mice only 1–2% circulate in the peripheral blood [10]. Release of neutrophils from the bone marrow depends on a series of stimulating factors and cytokines including IL-23, IL-17, G-CSF; and CXC chemokine receptors [11, 12]. The generation and maturation of neutrophils have important implications: from the design of therapeutic strategies to the utilization of their expression as a prognostic biomarker.

### Neutrophils role in cancer

**Fig. 1:**
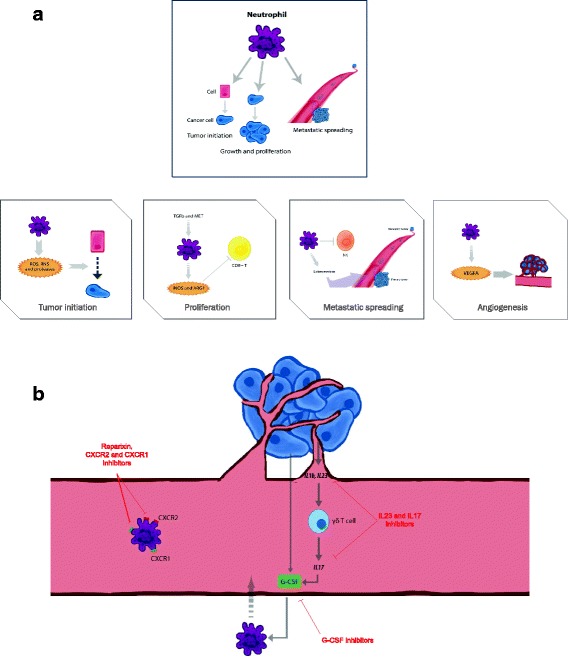
**a**. Mechanisms associated with the participation of neutrophils in the oncogenic process. Neutrophils are involved in various oncogenic processes such as tumor initiation, growth and proliferation, dissemination to other tissues, and formation of new blood vessels in the tumor. **b**. Therapeutic strategies to inhibit the oncogenic effect of neutrophils at different levels. Different compounds have been developed to target factors produced by the tumor and also to receptors present in neutrophils that favor the migration of neutrophils to the tumoral areas

The role of neutrophils in cancer is multifactorial and not fully understood. Neutrophils reflect a state of host inflammation, which is a hallmark of cancer [13]. They can participate in different stages of the oncogenic process including tumor initiation, growth, proliferation or metastatic spreading [8, 9]. In general neutrophils play a central role in inflammation within the tumor as they are attracted by CXCR2 ligands like CXCL1, CXCL2 and CXCL5, among others [9, 14]. Tumor initiation can be promoted by the release by neutrophils of reactive oxygen species (ROS), reactive nitrogen species (RNS) or proteases, among others [15]. A relevant mechanism is the induction of angiogenesis. Indeed, neutrophil depletion or CXCR2 blocking decrease vessel formation [15]. Some factors that mediate the formation of angiogenesis include the production of vascular endothelial growth factor A (VEGFA), prokineticin 2 (PROK2), or MMP9, among others [16, 17]. Neutrophils can facilitate tumor proliferation by attenuating the immune system. CD8+ T lymphocyte antitumor response can be suppressed by nitric oxide synthase (iNOS), or arginase 1 (ARG1) released by neutrophils under stimulation by TGFβ (Fig. 1a) [18, 19]. They also produce MMP9 that has an important role in tumor initiation. In addition tumor proliferation can be mediated by degradation of the insulin receptor substrate 1 (IRS1), and activation of PI3K signaling due to the transfer of neutrophil elastase to cancer cells [20]. Of note, production of iNOS can also be stimulated in neutrophils by the upregulation of the tyrosine kinase receptor MET [21]. Finally, neutrophils can also motivate the metastatic spreading by inhibiting natural killer function and facilitating the extravasation of tumor cells (Fig. 1a) [22, 23]. As can be seen here, the role of neutrophils in cancer is complex, and can be context and tumor dependent. Indeed, some studies have even shown how neutrophils can antagonize the metastatic spreading, as is the case in lung cancer [24]. It should be mentioned that this difference in function could be linked with the existence of various neutrophil subpopulations [8, 9].

A different population of cells that is generated in the bone marrow from myeloid precursors is the myeloid-derived suppressor cells (MDSC). They migrate to the tumor guided by several stimulating factors, being the chemokines CCL2 and CCL5 the most studied [25–27]. There are two different type of cells, polymorphonuclear MDSC (PMN-MDSC), that are morphologically similar to neutrophils, and monocytic MDSC (M-MDSC), that are similar to monocytes [27]. Of note, MDSC have a potent suppressor capacity in human cancer [27].

### Association of neutrophil presence and clinical outcome

Given the various roles of neutrophils in cancer development and progression, several groups have recently explored the role of neutrophils and other markers of host inflammation on clinical outcomes. Thus, an elevated neutrophil count is an adverse prognostic factor incorporated in a contemporary prognostic score for metastatic renal cell carcinoma (mRCC) treated with targeted therapy [28]. Furthermore, most data are available for the ratio of neutrophils to lymphocytes measured in the peripheral blood, the so-called neutrophil-to-lymphocyte ratio (NLR). An elevated NLR is associated with worse outcomes in many solid tumors, both in early and advanced stage of cancer [3]. Moreover, an elevated NLR is associated with lower response rates in castration-resistant prostate cancer treated with abiraterone or docetaxel [29, 30] and a decline during treatment with cabazitaxel was shown to be associated with longer overall survival [31]. Also, an early decrease of NLR in response to targeted treatment appears to be associated with more favorable outcomes and higher response rates in patients with mRCC, even after adjustment for known prognostic factors including NLR at baseline [5]. In contrast a rising NLR during the first weeks of treatment had the opposite effect. These findings make NLR a biomarker easy to evaluate, and that have potential for the identification of early responders. Table 1 summarizes all the meta-analyses studies performed evaluating the role of NLR expression and outcome in cancer.

**Table 1:** Overview of meta-analyses of the prognostic role of the neutrophil-to-lymphocyte ratio (NLR) in solid tumours

Reference					Prognostic outcome for high NLR: If not otherwise indicated, Hazard Ratio [95% confidence interval]			
PubMed identifier	Author	Tumor type	Number of studies	Number of patients	OS	PFS	DFS/RFS	EFS
PMID: 27,368,058	Cao J. et al.	Prostate cancer	22	18,092	1.40 [1.25–1.55]	1.42 [1.23–1.61]	1.38 [1.01–1.75]	-
PMID: 25,889,889	Chen J. et al.	Gastric cancer	9	3709	2.16 [1.86–2.50]	2.78 [1.95–3.96]	-	-
PMID: 28,430,605	Chen N. et al.	Malignant pleural mesothelioma	11	1533	1.48 [1.16–1.89]	-	-	-
PMID: 26,226,887	Cheng H. et al.	Pancreatic cancer	9	2035	1.59 [1.41–1.79]	-	-	-
PMID: 28,693,795	Dolan R.D. et al.	Advanced inoperable cancer	59	16,921	1.71 [1.57–1.86]	-	-	-
PMID: 28,222,899	Ethier J.L. et al.	Gynecologic cancer	26	10,530	1.65 [1.44–1.89]	-	-	1.57 [1.35–1.82]
PMID: 26,912,340	Gu X. et al.	Prostate cancer	14	16,266	1.38 [1.22–1.56]	1.24 [1.05–1.46]	-	-
PMID: 26,205,001	Gu X.B. et al.	Non- small cell lung cancer	14	3656	1.70 [1.39–2.09]	1.63 [1.27–2.09]	-	-
PMID: 25,854,964	Hu K. et al.	Renal cell carcinoma	15	3357	1.82 [1.51–2.19]	2.18 [1.75–2.71]	-	-
PMID: 28,467,978	Huang Q.T. et al.	Ovarian cancer	12	3854	1.69 [1.29–2.22]	1.63 [1.27–2.09]	-	-
PMID: 28,187,430	Huang Q.T. et al.	Cervical cancer	9	2804	1.88 [1.30–2.73]	1.65 [1.18–2.29]	-	-
PMID: 24,122,750	Li M.X. et al.	Colorectal cancer	16	6859	1.81 [1.50–2.19]	2.10 [1.55–2.84]	-	-
PMID: 28,514,738	Li X. et al.	Upper urinary tract and bladder	32	11,538	1.72 [1.45–2.05]	1.68 [1.44–1.96]	-	-
PMID: 26,835,589	Li Y. et al.	Soft tissue sarcoma	11	1809	3.75 [1.24–11.37]	-	2.43 [0.84 –7.05]	-
PMID: 26,448,011	Luo Y. et al.	Renal cell carcinoma	34	9811	1.79 [1.61–2.00]	1.85 [1.24–2.77]	1.97 [1.37–2,84]	-
		Upper tract urothelial carcinoma			2.48 [1.31–4.70]	1.70 [1.14–2.56]	1.47 [1.11–1.95]	-
		Bladder cancer			1.68 [1.45–1.94]	3.52 [1.33–9.33]	1.55 [1.21–2.00]	-
		Prostate cancer			1.44 [1.28–1.62]	1.29 [1.04–1.59]	-	-
PMID: 24,866,438	Malietzis G. et al.	Colorectal cancer	13	4056	-	-	2.08 [1.64–2.64]	-
PMID: 28,131,752	Marchioni M. et al.	Upper tract urothelial cancer	6	1710	1.97 [1.27–3.04]	-	1.53 [1.19–1.96]	-
PMID: 28,602,879	Mei Z. et al.	Advanced cancer	66	24,536	1.70 [1.57–1.84]	-	1.61 [1.42–1.82]	-
PMID: 27,270,655	Na N. et al.	Renal carcinoma	9	1091	1.93 [1.35–2.77]	2.12 [1.42–3.17]	-	-
PMID: 24,378,193	Paramanathan A. et al.	Solid tumors	49	14,282	1.92 [1.64–2.24]	-	1.99 [1.80–2.20]	-
PMID: 26,064,198	Peng B. et al.	Non-small cell lung cancer	12	2377	1.43 [1.25–1.64]	1.37 [1.07–1.74]	-	-
PMID: 28,296,774	Su L. et al.	Nasopharyngeal carcinoma	14	11,651	1.77 [1.41–2.23]	1.67 [1.36–2.06]	-	-
PMID: 26,924,872	Sun J. et al.	Gastric cancer	19	5431	1.98 [1.75–2.24]	1.58 [1.32–1.88]	-	-
PMID: 27,427,969	Tang H. et al.	Colorectal Liver metastasis	8	1685	2.17 [1.82–2.58]	-	1.96 [1.64–2.35]	-
PMID: 27,096,158	Tang L. et al.	Advanced Prostate cancer	18	9418	1.628 [1.41–1.879]	-	1.37 [1.13–1.64]	-
PMID: 24,875,653	Templeton A.J. et al.	Solid tumors	100	40,559	1.81 [1.67–1.97]	1.63 [1.39–1.91]	2.27 [1.85–2.79]	-
PMID: 27,461,614	Tsai P.L. et al.	Colorectal cancer	15	7741	OR: 2.03 [1.56–2.63]	-	OR: 1.67 [1.19–2.35]	-
PMID: 27,660,475	Wei B. et al.	Breast cancer	12	7951	2.03 [1.41–2.93]	-	1.46 [1.12–1.90]	-
PMID: 24,642,859	Wei Y. et al.	Urinary cancer	17	3159	1.81 [1.48–2.21]	-	2.07 [1.65–2.6]	-
PMID: 28,077,792	Wu J. et al.	Cervical cancer	13	3729	1.38 [1.20–1.58]	1.65 [1.31–2.07]	-	-
PMID: 24,559,042	Xiao W.K. et al.	Hepatocellular carcinoma	15	3094	3.42 [2.41–4.85]	-	5.90 [3.99–8.70]	-
PMID: 26,225,826	Xin-Ji Z. et al.	Gastric cancer	29	14,321	1.65 [1.47–1.83]	-	1.61 [1.28–1.94]	-
PMID: 24,788,770	Xue T.C. et al.	Liver cancer	26	4461	2.10 [1.74–2.54]	-	2.47 [1.85–3.30]	-
PMID: 27,732,958	Yang H.B. et al.	Lung cancer	19	7283	1.23 [1.17–1.29]	1.18 [1.08–1.29]	-	-
PMID: 25,759,553	Yang J.J. et al.	Pancreatic cancer	11	1804	2.61 [1.68–4.06]	-	-	-
PMID: 25,914,549	Yang X. et al.	Esophageal cancer	6	1633	1.54 [1.32–1.80]	-	1.74 [1.25–2.43]	-
PMID: 28,423,365	Yang Z. et al.	Epithelial ovarian cancer	12	3154	1.72 [1.18–2.51]	1.80 [1.22–2.65]	-	-
PMID: 26,817,900	Yin X. et al.	Prostate cancer	14	12,474	1.45 [0.77–2.71]	-	1.34 [0.89–2.02]	-
		Metastatic castration resistant prostate cancer			1.57 [1.41–1.74]	1.97 [1.28–3.04]	-	-
PMID: 26,222,823	Yin Y. et al.	Lung cancer	14	2734	1.51 [1.32–1.72]	-	-	-
PMID: 26,416,715	Yodying H. et al.	Esophageal cancer	7	1540	1.40 [1.08–1.81]	-	1.54 [0.79–2.98]	-
PMID: 28,644,143	Zhang J. et al.	Colorectal cancer	23	11,762	1.92 [1.57–2.34]	-	1.66 [1.31–2.11]	-
PMID: 25,401,500	Zhang X. et al.	Gastric cancer	10	2952	1.83 [1.62–2.07]	1.54 [1.22–1.95]	1.58 [1.12–2.21]	-
PMID: 26,491,346	Zhao Q.T. et al.	Lung cancer	22	7054	1.51 [1.33–1.71]	1.33 [1.07–1.67]	-	-

Abbreviations: *NLR* neutrophil-to-lymphocyte ratio, *DFS/RFS* Disease-free survival/Recurrence-free survival, *PFS* Progression-free survival, *OS* Overall survival, *EFS* Event-free survival, *OR* Odds ratio

Not only elevated numbers of neutrophils in peripheral blood as reflected by NLR are of prognostic relevance, but also their presence in the tumor can be associated with clinical outcome. The expression of neutrophils in the tumor has been linked with detrimental outcome in some indications like in renal cell carcinoma, head and neck cancer or esophageal carcinoma [6, 32, 33]; whereas in other indications it has been associated with better survival [34, 35]. In this context, it should be noted that what mainly impact the worse outcome is the presence of inflammation within the tumor, and the assessment of neutrophils is an indirect measure of this and can vary among tumor types.

### Therapeutic strategies to decrease neutrophil activity

To avoid the deleterious effect of neutrophil expression in cancer, strategies intended to reduce its activity have been explored and some have entered clinical evaluation. Table 2 describes characteristics of all ongoing clinical studies. The first approach is to target factors involved in the late stage process of neutrophil maturation. Indeed, some factors can be produced by tumor cells and this may favor the metastatic spreading mediated by neutrophils (Fig. 1b) [36, 37].

**Table 2:** List of compounds and targets that are currently in clinical development

Drug	Mechanism of action	Study number	Clinical stage	Indication	Alone or in combination
Reparixin	Reparixin Noncompetitive allosteric inhibitor of CXCR1 and CXCR2 chemokine.	NCT02001974	I	Metastatic Breast Cancer	Paclitaxel + Reparixin
Reparixin	Reparixin Noncompetitive allosteric inhibitor of CXCR1 and CXCR2 chemokine	NCT01861054	II	Metastatic Breast Cancer	Alone
Reparixin	Reparixin Noncompetitive allosteric inhibitor of CXCR1 and CXCR2 chemokine	NCT02370238	II	Metastatic Breast Cancer	Paclitaxel in Combination With Reparixin or Placebo

Strategies explored to inhibit neutrophils include the inhibition of CXC receptors like CXCR2 that are associated with the migration of neutrophils to tumor areas. CXCR1 and CXCR2 inhibitors are currently in clinical development in cancer [38, 39]. Inhibition of the IL-23 and IL-17 axis is another approach, as IL-17 and IL-23 stimulate expansion of neutrophils mediated by G-CSF (Fig. 1b) [40]. However this approach has not reached yet the oncology field, but drugs targeting these cytokines are approved for the treatment of other medical conditions like psoriasis [41, 42].

Another tactic is to directly inhibit G-CSF and therefore decrease the amount of neutrophils, strategy that has shown efficacy in preclinical models [43]. Agents against this target are currently in its early stage of clinical development in cancer [44]. However, it is unclear if the inhibition of G-CSF and subsequent reduction of neutrophils can have an impact in patient infections, mainly in those under treatment with chemotherapy. Recently, preclinical studies have shown that neutrophil Alox5 inhibition can also decrease metastatic lung dissemination (Fig. 1b) [45].

### Next steps

There are many areas of uncertainty regarding the evaluation of neutrophils as a prognostic marker or in the development of compounds against neutrophils.

Although the NLR is considered as an easy, inexpensive and reproducible biomarker associated with clinical outcome for the majority of tumors some questions remain to be resolved. For instance, the identification of adequate cut-offs, or longitudinal evaluations over a treatment period of time could add more accurate information. Indeed, modifications over time can inform about treatment efficacy. Similarly, comparison of this ratio with the expression of cytokines in blood or the evaluation of neutrophil expression in tumors could help to improve its prognostic or predictive value.

It is also challenging how to optimize therapies against neutrophils. Some studies have suggested an augmented effect when neutrophil targeting agents, CXCR2 inhibitors or anti-Ly6G, were combined with checkpoint inhibitors [46, 47]. Table 2 provides a list of compounds in clinical development. Similarly combinations of antiangiogenic agents with neutrophil targeting agents could be another tactic as resistance to antiangiogenic agents has been linked with neutrophil stimulation [48]. In the case of combination strategies with chemotherapy, data is contradictory with studies supporting the efficacy of the combination and others showing a detrimental effect [49]. Of note clinical studies in combination with chemotherapy are also present. Like with any new therapeutic agent, identification of a biomarker or a specific clinical scenario could undoubtedly help to identify responsive patients. Finally, given the dual role of neutrophils in cancer, the consequences of depleting tumor promoting and anti-tumor neutrophils are unclear, reinforcing the importance for patient identification and biomarker discovery.

## Conclusion

In conclusion, neutrophils are new players in cancer and have a potential role as biomarkers of disease outcome or as therapeutic targets. However, there is still much work to be done before they might be used as validated prognostic markers, or agents against them will reach the clinical setting.

## References

[1] Ocana A, Pandiella A, Siu LL, Tannock IF (2010). Preclinical development of molecular-targeted agents for cancer. Nat Rev Clin Oncol.

[2] Atkins MB, Larkin J (2016). Immunotherapy combined or sequenced with targeted therapy in the treatment of solid tumors: current perspectives. J Natl Cancer Inst.

[3] Templeton AJ, McNamara MG, Seruga B (2014). Prognostic role of neutrophil-to-lymphocyte ratio in solid tumors: a systematic review and meta-analysis. J Natl Cancer Inst.

[4] van Soest RJ, Templeton AJ, Vera-Badillo FE (2015). Neutrophil-to-lymphocyte ratio as a prognostic biomarker for men with metastatic castration-resistant prostate cancer receiving first-line chemotherapy: data from two randomized phase III trials. Ann Oncol.

[5] Templeton AJ, Knox JJ, Lin X (2016). Change in Neutrophil-to-lymphocyte ratio in response to targeted therapy for metastatic renal cell carcinoma as a prognosticator and biomarker of efficacy. Eur Urol.

[6] Jensen HK, Donskov F, Marcussen N (2009). Presence of intratumoral neutrophils is an independent prognostic factor in localized renal cell carcinoma. J Clin Oncol.

[7] Bronte V, Brandau S, Chen SH (2016). Recommendations for myeloid-derived suppressor cell nomenclature and characterization standards. Nat Commun.

[8] Swierczak A, Mouchemore KA, Hamilton JA, Anderson RL (2015). Neutrophils: important contributors to tumor progression and metastasis. Cancer Metastasis Rev.

[9] Coffelt SB, Wellenstein MD, de Visser KE (2016). Neutrophils in cancer: neutral no more. Nat Rev Cancer.

[10] Rosenbauer F, Tenen DG (2007). Transcription factors in myeloid development: balancing differentiation with transformation. Nat Rev Immunol.

[11] McKinstry WJ, Li CL, Rasko JE (1997). Cytokine receptor expression on hematopoietic stem and progenitor cells. Blood.

[12] Steinbach KH, Schick P, Trepel F (1979). Estimation of kinetic parameters of neutrophilic, eosinophilic, and basophilic granulocytes in human blood. Blutalkohol.

[13] Hanahan D, Weinberg RA (2011). Hallmarks of cancer: the next generation. Cell.

[14] Jamieson T, Clarke M, Steele CW (2012). Inhibition of CXCR2 profoundly suppresses inflammation-driven and spontaneous tumorigenesis. J Clin Invest.

[15] Antonio N, Bonnelykke-Behrndtz ML, Ward LC (2015). The wound inflammatory response exacerbates growth of pre-neoplastic cells and progression to cancer. EMBO J.

[16] Shojaei F, Wu X, Zhong C (2007). Bv8 regulates myeloid-cell-dependent tumour angiogenesis. Nature.

[17] Shojaei F, Singh M, Thompson JD, Ferrara N (2008). Role of Bv8 in neutrophil-dependent angiogenesis in a transgenic model of cancer progression. Proc Natl Acad Sci U S A.

[18] Fridlender ZG, Sun J, Kim S (2009). Polarization of tumor-associated neutrophil phenotype by TGF-beta: “N1” versus “N2” TAN. Cancer Cell.

[19] Bodogai M, Moritoh K, Lee-Chang C (2015). Immunosuppressive and Prometastatic functions of myeloid-derived suppressive cells rely upon education from tumor-associated B cells. Cancer Res.

[20] Houghton AM, Rzymkiewicz DM, Ji H (2010). Neutrophil elastase-mediated degradation of IRS-1 accelerates lung tumor growth. Nat Med.

[21] Finisguerra V, Di Conza G, Di Matteo M (2015). MET is required for the recruitment of anti-tumoural neutrophils. Nature.

[22] Welch DR, Schissel DJ, Howrey RP, Aeed PA (1989). Tumor-elicited polymorphonuclear cells, in contrast to “normal” circulating polymorphonuclear cells, stimulate invasive and metastatic potentials of rat mammary adenocarcinoma cells. Proc Natl Acad Sci U S A.

[23] Spiegel A, Brooks MW, Houshyar S (2016). Neutrophils suppress Intraluminal NK cell-mediated tumor cell clearance and enhance Extravasation of disseminated carcinoma cells. Cancer Discov.

[24] Granot Z, Henke E, Comen EA (2011). Tumor entrained neutrophils inhibit seeding in the premetastatic lung. Cancer Cell.

[25] Qian BZ, Li J, Zhang H (2011). CCL2 recruits inflammatory monocytes to facilitate breast-tumour metastasis. Nature.

[26] Huang B, Lei Z, Zhao J (2007). CCL2/CCR2 pathway mediates recruitment of myeloid suppressor cells to cancers. Cancer Lett.

[27] Kumar V, Patel S, Tcyganov E, Gabrilovich DI (2016). The nature of myeloid-derived suppressor cells in the tumor microenvironment. Trends Immunol.

[28] Heng DY, Xie W, Regan MM (2009). Prognostic factors for overall survival in patients with metastatic renal cell carcinoma treated with vascular endothelial growth factor-targeted agents: results from a large, multicenter study. J Clin Oncol.

[29] Leibowitz-Amit R, Templeton AJ, Omlin A (2014). Clinical variables associated with PSA response to abiraterone acetate in patients with metastatic castration-resistant prostate cancer. Ann Oncol.

[30] Templeton AJ, Pezaro C, Omlin A (2014). Simple prognostic score for metastatic castration-resistant prostate cancer with incorporation of neutrophil-to-lymphocyte ratio. Cancer.

[31] Lorente D, Mateo J, Templeton AJ (2015). Baseline neutrophil-lymphocyte ratio (NLR) is associated with survival and response to treatment with second-line chemotherapy for advanced prostate cancer independent of baseline steroid use. Ann Oncol.

[32] Trellakis S, Bruderek K, Dumitru CA (2011). Polymorphonuclear granulocytes in human head and neck cancer: enhanced inflammatory activity, modulation by cancer cells and expansion in advanced disease. Int J Cancer.

[33] Wang J, Jia Y, Wang N (2014). The clinical significance of tumor-infiltrating neutrophils and neutrophil-to-CD8+ lymphocyte ratio in patients with resectable esophageal squamous cell carcinoma. J Transl Med.

[34] Caruso RA, Bellocco R, Pagano M (2002). Prognostic value of intratumoral neutrophils in advanced gastric carcinoma in a high-risk area in northern Italy. Mod Pathol.

[35] Galdiero MR, Bianchi P, Grizzi F (2016). Occurrence and significance of tumor-associated neutrophils in patients with colorectal cancer. Int J Cancer.

[36] Waight JD, Hu Q, Miller A (2011). Tumor-derived G-CSF facilitates neoplastic growth through a granulocytic myeloid-derived suppressor cell-dependent mechanism. PLoS One.

[37] Kowanetz M, Wu X, Lee J (2010). Granulocyte-colony stimulating factor promotes lung metastasis through mobilization of Ly6G+Ly6C+ granulocytes. Proc Natl Acad Sci U S A.

[38] Bertini R, Allegretti M, Bizzarri C (2004). Noncompetitive allosteric inhibitors of the inflammatory chemokine receptors CXCR1 and CXCR2: prevention of reperfusion injury. Proc Natl Acad Sci U S A.

[39] Baselga J, Tabernero JM (2001). Weekly docetaxel in breast cancer: applying clinical data to patient therapy. Oncologist.

[40] Stark MA, Huo Y, Burcin TL (2005). Phagocytosis of apoptotic neutrophils regulates granulopoiesis via IL-23 and IL-17. Immunity.

[41] Gaffen SL, Jain R, Garg AV, Cua DJ (2014). The IL-23-IL-17 immune axis: from mechanisms to therapeutic testing. Nat Rev Immunol.

[42] Mei J, Liu Y, Dai N (2012). Cxcr2 and Cxcl5 regulate the IL-17/G-CSF axis and neutrophil homeostasis in mice. J Clin Invest.

[43] Ries CH, Cannarile MA, Hoves S (2014). Targeting tumor-associated macrophages with anti-CSF-1R antibody reveals a strategy for cancer therapy. Cancer Cell.

[44] Cassier PA, Italiano A, Gomez-Roca CA (2015). CSF1R inhibition with emactuzumab in locally advanced diffuse-type tenosynovial giant cell tumours of the soft tissue: a dose-escalation and dose-expansion phase 1 study. Lancet Oncol.

[45] Wculek SK, Malanchi I (2015). Neutrophils support lung colonization of metastasis-initiating breast cancer cells. Nature.

[46] Highfill SL, Cui Y, Giles AJ (2014). Disruption of CXCR2-mediated MDSC tumor trafficking enhances anti-PD1 efficacy. Sci Transl Med.

[47] Kim K, Skora AD, Li Z (2014). Eradication of metastatic mouse cancers resistant to immune checkpoint blockade by suppression of myeloid-derived cells. Proc Natl Acad Sci U S A.

[48] Chung AS, Wu X, Zhuang G (2013). An interleukin-17-mediated paracrine network promotes tumor resistance to anti-angiogenic therapy. Nat Med.

[49] Coffelt SB, de Visser KE (2015). Immune-mediated mechanisms influencing the efficacy of anticancer therapies. Trends Immunol.

